# An Investigation of the Dosimetric Uncertainties in the myOSLchip System: The Impact of Contributing Factors on Measurement Accuracy

**DOI:** 10.7759/cureus.88239

**Published:** 2025-07-18

**Authors:** Lee C Goddard, Amar Basavatia, Patrik Brodin, Wolfgang A Tomé

**Affiliations:** 1 Radiation Oncology, Montefiore Medical Center, Bronx, USA; 2 Institute for Onco-Physics, Albert Einstein College of Medicine, Bronx, USA

**Keywords:** clinical validation, energy dependence, in-vivo dosimetry, measurement accuracy, myosld, osld

## Abstract

The use of in vivo dosimetry (IVD) is integral to radiation oncology, particularly for treatments involving custom boluses or superficial electron lesions. In this report, the “myOSLchip” system, comprising beryllium oxide (BeO)-based optically stimulated luminescent chips and hand-held readers, was evaluated for clinical use. Each 4.7 × 0.5 mm chip, housed in plastic with an effective measurement point 1 mm below the surface, was calibrated under standard 6 megavoltage (MV) photon conditions using a 10 × 10 cm² field at 100 cm SSD and 1.5 cm depth in solid water. Calibration factors were assessed for energy dependence, dose linearity, time to readout, repeat measurements, and angular dependence. Results showed high measurement accuracy and linearity up to 10 Gy, with separate calibration factors needed per reader. The chips exhibited significant under-response at low energies (down to 16.82 keV), but minor variation in the megavoltage range for both photons and electrons. Repeat use showed an average signal loss of 1.2% after the first bleaching and 0.35% per subsequent cycle. A stable readout required at least four hours post-irradiation, with earlier readings (30-60 minutes) showing 2-3% error. No angular dependence was observed. With appropriate calibration and consideration of these factors, the myOSLchip system delivers dose measurements within the clinically accepted 3-5% accuracy range.

## Introduction

In vivo dosimetry (IVD) is the process of measuring radiation dose directly within or on the surface of the patient during radiation therapy to ensure the accurate delivery of the prescribed dose. IVD serves as a critical quality assurance tool, verifying that the dose delivered to the patient matches the intended dose. Deviations in delivered dose can significantly impact treatment outcomes, potentially compromising tumor control or increasing the risk of normal tissue complications [1]. The need for robust IVD arises from the complexity of modern treatment techniques, such as intensity-modulated radiation therapy (IMRT) and volumetric modulated arc therapy (VMAT), where steep dose gradients and highly conformal dose distributions demand precise monitoring [2]. Even relatively simple treatment techniques can have deviations in the delivered dose, due to variations in patient positioning, anatomical changes, and uncertainties in beam delivery.

Optically stimulated luminescence dosimeters (OSLDs) are devices used to measure radiation dose by utilizing optically stimulated luminescence (OSL) [3]. This process involves trapping energy from ionizing radiation within a sensitive material, which is later released as light when stimulated by a specific wavelength of light. The emitted luminescence is directly proportional to the absorbed radiation dose, making OSLDs highly dependable for precise dose measurement. The presence of impurities within the sensitive material can lead to low-energy traps located close to the conduction band. These low-energy traps are thermally unstable, allowing trapped electrons to escape even at room temperature. This spontaneous release of charge leads to signal fading, particularly when there is a delay between irradiation and readout. Repeated readout and erasure of the sensitive material can also affect the sensitivity of OSLD materials.

The “myOSLchip” dosimeter system (RadPro International GmbH, Remscheid, Germany) consists of the myOSLchip dosimeter (“chip”) and a handheld OSL reader. The active region of the chip consists of beryllium oxide (BeO) semiconductor material with a density of ~3 gcm^-3^ and an effective atomic number (Zeff) of ~7.2. BeO has been utilized in both thermo-luminescent dosimeters (TLDs) and OSLDs [4]. BeO has been shown to have a linear response when exposed to doses of up to ~ 10 Gy; however, a large variance has been noted between individual chips as well as a decline in sensitivity when repeated irradiation/bleaching cycles are performed [5].

The American Association of Physicists in Medicine Task Group 191 (AAPM TG 191) provides guidelines for the use of OSLDs in a clinical setting [4]; however, it mainly focuses on the TLD-100 (Thermo Fischer Scientific, Waltham, MA) and now-recalled nanoDot (Landauer Inc., Glenwood, IL) systems. We performed a comprehensive evaluation of the dosimetric measurement accuracy and energy dependence of the myOSLchip BeO dosimeters in the context of clinical IVD according to the processes of the AAPM TG 191 report. During the course of this work, other groups have published their own independent evaluations of the MyOSLChip, most notably the work of Kowalski et al. [6]. This work provides both a confirmation of their findings with multiple readers and an expansion of their findings with regard to the energy dependence of the myOSLchip below the megavoltage (MV) range.

## Technical report

Materials and methods

myOSLchip System and Reader Setup

The myOSLchip OSLD system is designed for accurate radiation dose measurement in clinical and research settings. The chip consists of a BeO semiconductor (4.7 mm square, 0.5 mm thick) enclosed in a plastic case (9.5 mm x 10.5 mm, 2 mm thick). The effective point of measurement is 1 mm below the surface. Each chip has an identifying number and QR code printed on the surface to enable identification by the user and within the OSL reader. The chips are reusable and can store dose information until readout, allowing flexibility in clinical workflows.

The reader device stimulates the OSLD chips using a blue LED light source (~460 nm) and measures the resulting luminescence. The reader is also capable of bleaching the chip using continued exposure to a separate high-power erase LED; however, the manufacturer states that this feature is not recommended for high-dose signals. There is a separate eraser unit available, which allows for the bleaching of up to 48 chips simultaneously and is recommended for use when erasing high-dose signals. The myOSLchip system has been characterized for use in radiation oncology and is a clinically feasible IVD system [7]. There are, however, a number of factors that must be taken into account to ensure accurate dose measurements. In this study, three myOSLchip readers were characterized independently for use at three clinical locations.

Light Counts (LC) per Gy at Reference Condition

To allow for consistent positioning of the chips in the irradiation beam and to minimize air gaps surrounding the chips, a 3D printed holder was created that allowed simultaneous irradiation of five chips. Each chip is located at the edge of a pentagon centered 15 mm from the isocenter. The holder was placed on 10 cm of “solid water” (Sun Nuclear, Melbourne, FL) to allow for sufficient backscatter, with 1.4 cm of solid water placed on top of the holder. The center of each myOSLchip was thus at a depth of 1.5 cm, the depth of maximum dose (dmax) for the 6 MV photon beam that was used to irradiate the chips. A 10 x 10 reference field was utilized with 100 cm SSD set to the surface of the solid water. Before irradiation, beam output was measured as specified in AAPM TG51, and the precise dose per monitor unit was measured for each LINAC utilized for calibration. After irradiation, 15 hours were allowed to pass before reading each chip. Three measurements were taken, and the average light count was utilized to calculate the calibration factor of LC per Gy for a given myOSLchip.

Energy Dependence

To examine the energy dependence of the chips in the MV range, a C-arm Truebeam LINAC (Varian, Palo Alto, CA) was utilized. Before irradiation, output measurements were performed for five photon energies and four electron energies, per AAPM TG51. Before irradiation with different energies, a chip-specific LC per Gy factor was determined in a 6 MV beam, as outlined above. Once these chip-specific factors had been determined, the chips were erased before energy dependence measurements were acquired. For energy dependence exposures, a single chip was placed at isocenter with 10 cm of solid water backscatter and a 0.5 cm sheet of elastic gel bolus “superflab” backscatter (Radiation Products Design, Inc., Albertville, MN), to minimize air gaps around the chip. Solid water was placed on top of the chip/superflab such that the center of the chip was located at the depth of dmax for each energy utilized. The surface of the solid water was set to 100 cm SSD, and a 10 cm x 10 cm field was utilized for irradiation. Three chips were irradiated with 100 monitor units (MU) for each energy. After irradiation, 15 hours were allowed to pass before reading each chip. Three measurements were taken for each chip, and the average light count was utilized to calculate the dose measured with each chip for inter-energy comparison.

A further range of energies was used to characterize the response below the MV range. The Small Animal Radiation Research Platform (SARRP, Xstrahl, Suwanee, GA) was utilized to irradiate chips to known doses with beam energies ranging from 50 to 220 kVp. Known dose outputs were confirmed based on an in-house developed film calibration system [8]. Further energy dependence measurements were performed using the CIX-3 cabinet (Xstrahl) at 300 kVp using three different filtrations.

To complete the range of energies studied, an Ir-192 brachytherapy source (Flexitron, Elekta, Stockholm, Sweden) was used to determine the response at 365 keV, and a Shepard Mark-I Cesium irradiator (J L Shepherd & Associates, San Fernando, CA) was used to determine the response at 662 keV.

Calibration and Correction Factors

Other than the energy dependence, the factors considered for characterizing the uncertainty in the IVD readings using the myOSLchip were dose linearity, erasure correction, re-read correction, time to readout, and angular dependence.

Linearity was assessed by exposing 15 chips to increasing doses up to 12 Gy. Three different readers were utilized to evaluate differences between readers, with five chips being utilized per reader. Once exposed to a specified number of monitor units, 10 minutes were allowed to pass before each chip was read, and then exposed to the next incremental dose, and so on until all specified doses were recorded. After the final measurement, ten consecutive measurements were performed for each chip to allow for a re-read depletion correction to be determined. The average re-read correction factor, RCorrected, for each chip was found and utilized to make chip-specific corrections to each of the incremental dose readings.

To examine the effects of varying readout time, measurements were taken at different time intervals to allow for a read time correction curve to be generated. Finally, after the final readout at 16 hours, six consecutive measurements were performed for each chip to allow for a re-read depletion correction to be determined. The average re-read correction factor for each chip was found and utilized to make chip-specific corrections to each of the time-dependence readings.

Similarly, erasure correction was tested by exposing 36 chips to a known dose. After each exposure, 4 hours were allowed to pass before each chip was read. Two different readers were utilized, with 18 chips being utilized per reader. Three measurements were taken, and the average LC per Gy was determined. All 36 chips were erased utilizing the myOSL erasure unit, and consecutive cycles of bleaching and re-irradiation were examined.

$LC_{i}^{c} = \left\{ \begin{array}{ll} LC_{1}^{m} & \mathrm{for}\ i = 1 \\ LC_{i}^{m} - \left(1 - r_{c} \right) LC_{i-1}^{m} + LC_{i-1}^{c} & \mathrm{for}\ i > 1 \end{array} \right. \tag{1}$

where* LC_i_^C^* denotes the corrected light counts, *LC_i_^m^* the measured light counts, and *r_c_* denotes the read-read depletion correction. Equation 1 shows the re-read correction formula utilized to correct the measured values. The correction should be applied to the measured value and each sequential measurement.

Results

Dose Linearity Correction (k_L_)

**Figure 1:**
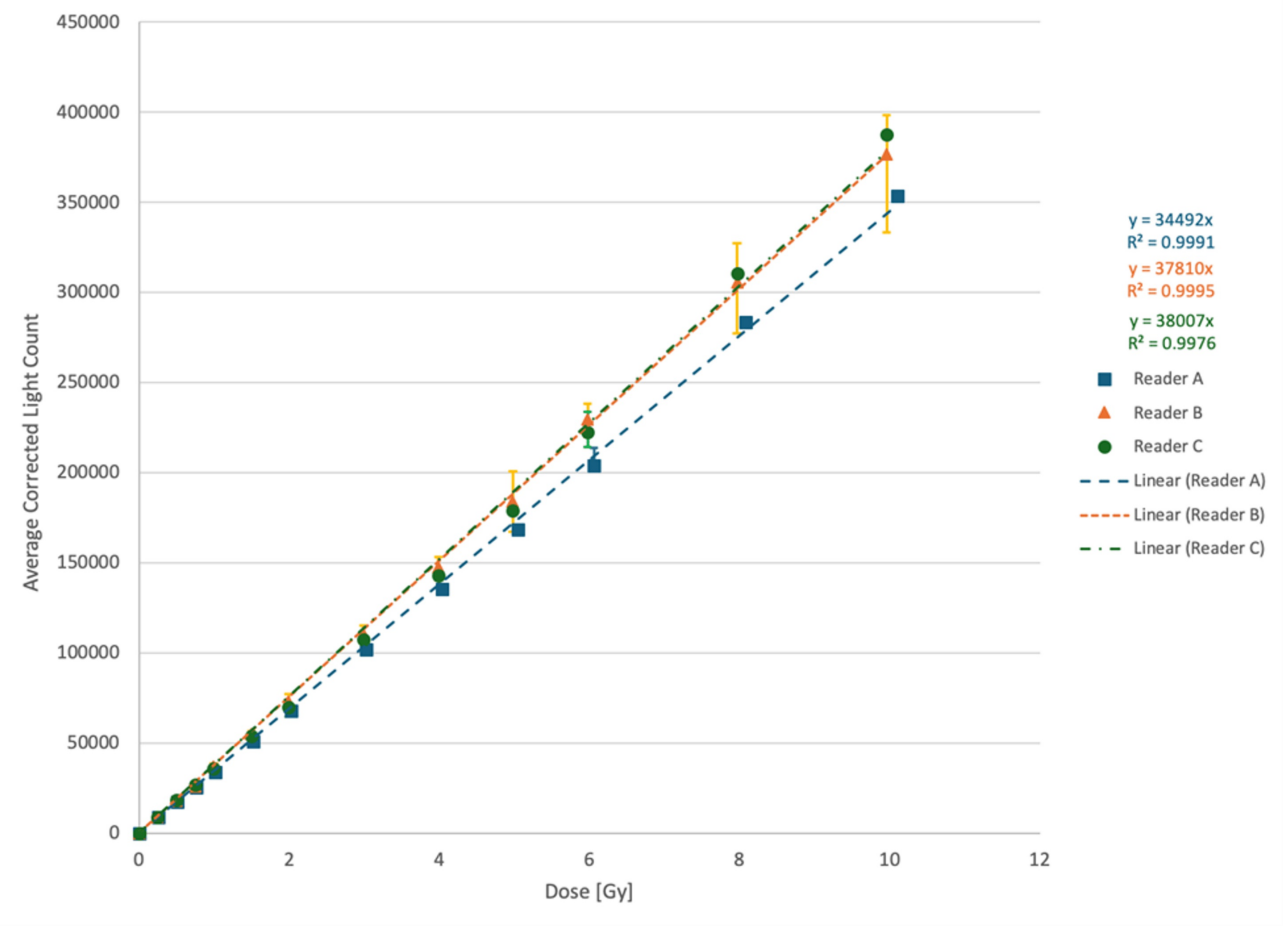
Light counts for three different readers as a function of delivered radiation dose Filled symbols represent average values, and uncertainty bars represent the range of all chips exposed to the given radiation dose Gy: Gray

The linearity in light count response was investigated for three different readers with delivered radiation doses up to 10 Gy. As shown in Figure 1, the response was linear up to the investigated dose of 10 Gy, and hence, a single LC/Gy calibration factor can be assigned to a given chip and reader combination. It is important to note that each reader should be characterized individually, as the response will not be the same between readers. Of note, the large uncertainty bars for reader B were due to a malfunction of the reader, which led to fluctuating readouts when re-reading chips multiple times instead of monotonically decreasing ones. This reader was returned to the manufacturer upon this discovery and replaced with a new reader.

Fading Correction (k_F_)

**Figure 2:**
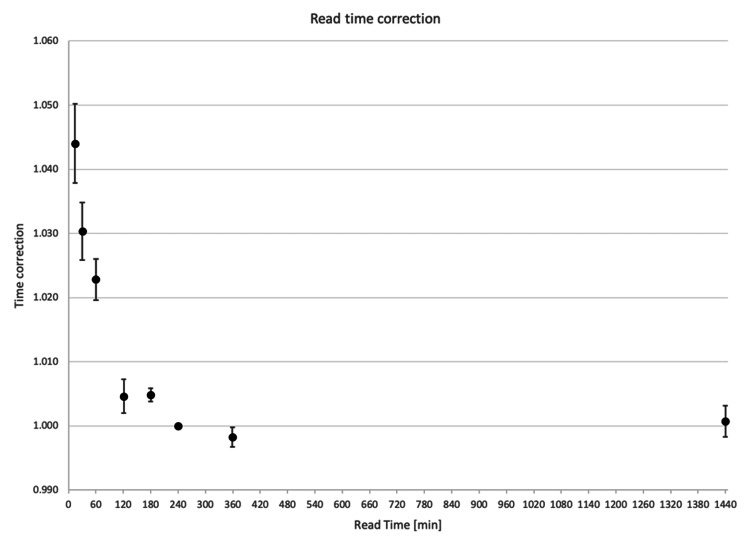
Light counts (LC) per Gy of delivered radiation dose as a function of time after irradiation to readout, normalized to the readings at 240 min Uncertainty bars represent 1 standard deviation

The LC per Gy of delivered dose was compared for chips readout at different times after irradiation, normalized to the reading at 240 min. Figure 2 demonstrates that for readouts less than four hours from irradiation, there is an over-response in the LC/Gy, whereas the response appears to stabilize for readings taken at longer than four hours.

Erasure Correction (k_ε_)

**Figure 3:**
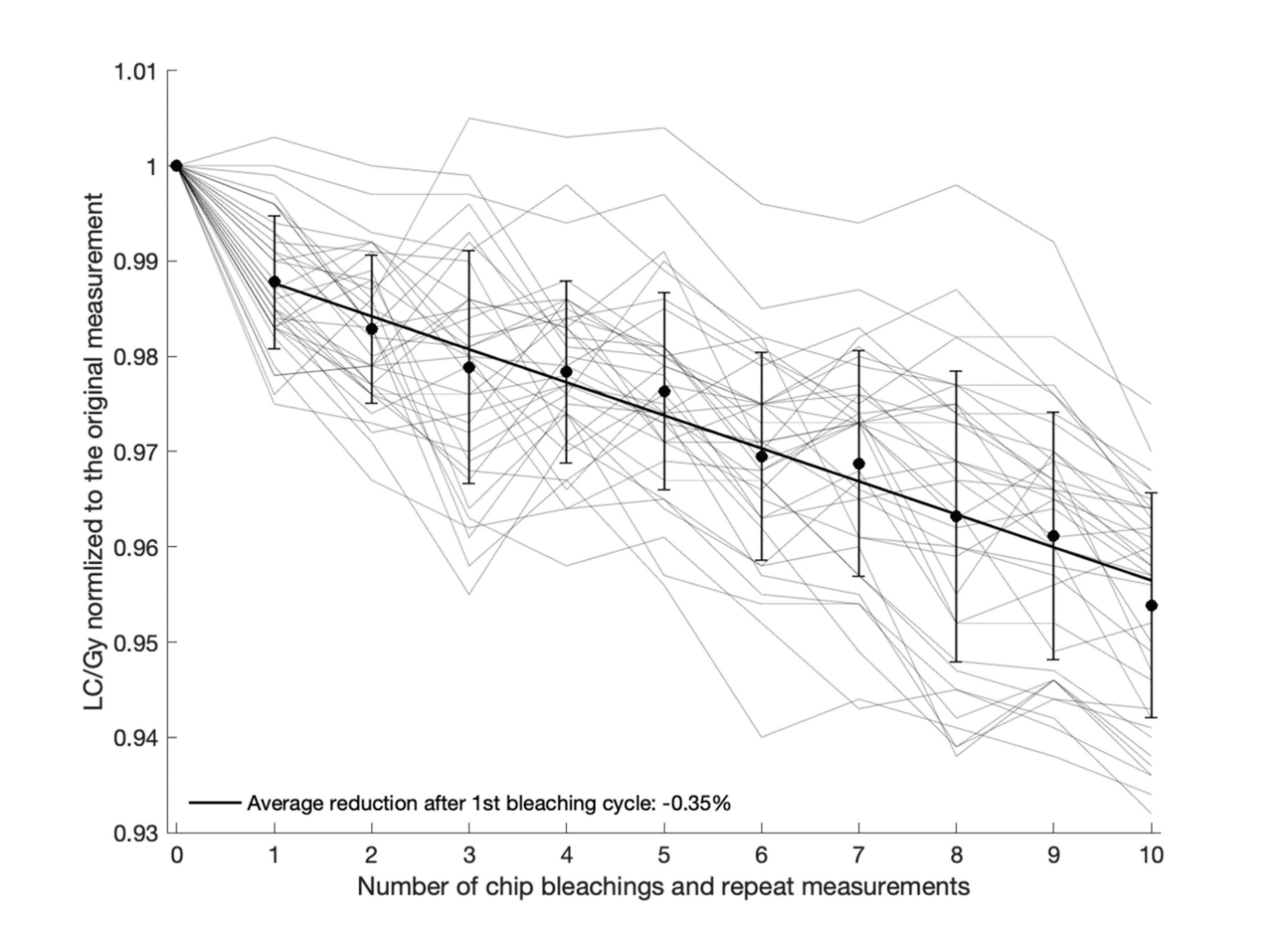
Light counts per Gy of delivered radiation dose as a function of the number of bleaching cycles and repeat measurements Thin shaded lines represent repeat measurement data of individual chips. Filled black circles with uncertainty bars represent the average and standard deviation of all chips at each repetition number, and the thick black line represents a linear fit of the average data points LC/Gy: light counts per Gray

Repeat IVD measurements can be performed by erasing, or bleaching, a chip after irradiation, essentially resetting it. Figure 3 shows the reduction in response with an increased number of repeated measurements evaluated on 36 individual chips. The first bleaching has the largest impact on chip response, with an average 1.2% reduction in LC/Gy, followed by an average 0.35% decline per bleaching cycle after that, although as illustrated, there is considerable variation between individual chips and from cycle to cycle.

Re-read Correction (k_ρ_)

The re-read correction was determined as the loss in signal from consecutively reading the same chip multiple times following a single irradiation. Thirty-six chips were used for this test, with each chip read a total of six times at 16 hours after irradiation. On average, between all chips, there was a 1.33% loss of signal with each repeat reading, and the average signal loss per repeat ranged from 0.95% - 1.74% between different chips.

Beam Quality Correction (k_Q_)

**Figure 4:**
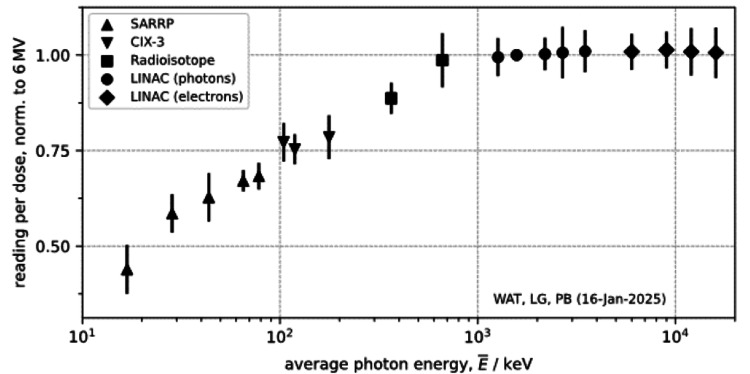
Energy dependence of the myOSLchip shown as a function of the mean energy of the delivered radiation quality Uncertainty bars represent 1 standard deviation LINAC: linear accelerator; SARRP: small animal radiation research platform; keV: kilo-electron volt

Due to the BeO material in the myOSLchip, they are expected to under respond at lower energies. Figure 4 illustrates this energy dependence as LC in the readout per Gy of delivered radiation dose, normalized to that of 6 MV photons. To generalize the interpretability of the results, the peak energy was given along with the mean energy of each spectrum, computed from the corresponding photon spectra, with details for each provided in Table 1. The results of the energy dependence evaluation clearly demonstrate the under-response at mean energies below 1000 keV, whereas for all clinical MV energies, the response is close to the same as for 6 MV photons.

**Table 1:** Energy dependence of the myOSLchip calibration coefficient characterized over a broad range of clinical and pre-clinical radiation qualities ^*^Mean Energy was computed from spectra generated using SpekPy Web version 2.08 [9] using the indicated tube potential, filtrations, SpekCalc Physics Model, and NIST XCOM attenuation data. ^#^Mean energy of photon beams was computed using PRIMO_Spectra_MOESM2_ESM data provided as supplementary data in Brualla et al. [10] LC/Gy: light counts per Gray; BE: beryllium; Al: aluminum; Cu: copper; Sn: tin; Ir: iridium; Cs: cesium; kVp: kilo-voltage peak; keV: kilo-electron volt; MV: megavoltage; FFF: flattening filter free; MeV: mega-electron volt

E_max_	Radiation quality	E_mean _[keV]	LC/Gy normalized to LC/Gy for 6 MV	Standard deviation
50 kVp	0.8 mm Be	16.82^*^	0.4390	0.0213
50 kVp	0.8 mm Be + 1 mm Al	28.45^*^	0.5853	0.0167
100 kVp	0.8 mm Be + 1 mm Al	43.59^*^	0.6276	0.0210
150 kVp	0.8 mm Be + 0.15 mm Cu	65.15^*^	0.6708	0.0092
220 kVp	0.8 mm Be + 0.15 mm Cu	78.05^*^	0.6830	0.0115
300 kVp	4.0 mm Be + 0.5 mm CU + 3 mm solid water	104.32^*^	0.7718	0.0167
300 kVp	4.0 mm Be + 1.0 mm CU+ 3 mm solid water	118.72^*^	0.7536	0.0129
300 kVp	4.0 mm Be + 2.0 mm CU + 2.0 mm AL + 1.5 mm Sn + 3 mm solid water	176.82^*^	0.78510	0.0190
612 keV	Ir-192 photons	365	0.8866	0.0135
662 keV	Cs-137 photons	662	0.9862	0.0236
6 FFF	Photons	1265^#^	0.9945	0.0165
6 MV	Photons	1571^#^	1.0	N/A
10 FFF	Photons	2186^#^	1.0029	0.0141
10 MV	Photons	2685^#^	1.0062	0.0224
15 MV	Photons	3489^#^	1.0097	0.0183
6 MeV	Electrons	6000	1.0087	0.0157
9 MeV	Electrons	9000	1.0132	0.0159
12 MeV	Electrons	12000	1.0085	0.0206
16 MeV	Electrons	16000	1.0062	0.0219

Angular Dependence (k_Θ_)

**Figure 5:**
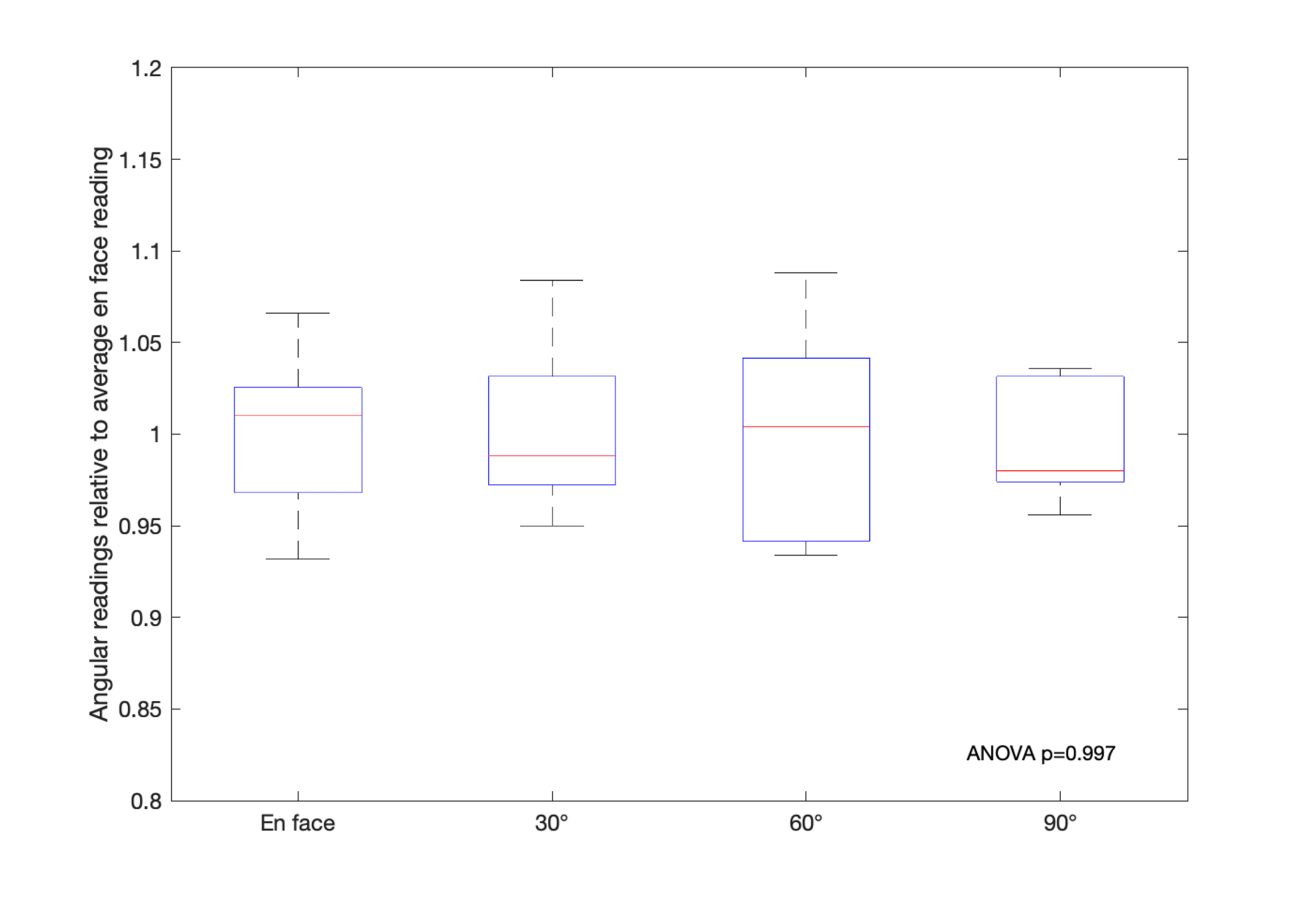
Angular readings relative to the average on face reading for multiple irradiation angles for five chips at each measurement angle Chip readout was performed 10 min following exposure

Attempts at characterizing any potential angular dependence of the myOSLchip were performed by irradiating and bleaching five chips, first in an en-face orientation facing the flat side of the chips, and then comparing to exposures from 30, 60, and 90 degrees with the chips placed in different orientations, allowing for measurements from all angles. There was no systematic angular dependence detected (p=0.997) beyond the intrinsic uncertainty in the measured dose, as the readings from varying angles seemed to vary randomly compared to the en-face irradiation, as shown in Figure 5.

Table 2 shows a summary of the measured correction factors and their impact on the dosimetric uncertainties of the myOSL chip system.

**Table 2:** Summary table of correction factors and their impact

Dose linearity correction (k_L_)	R^2^ ≥0.9976 up to 10 Gy for all three readers examined
Fading correction (k_F_)	~+5% at 10 minutes (relative to 4 hours)
Erasure correction (k_ε_)	1.2% at first bleaching cycle, 0.35% per subsequent cycle
Re-read correction (k_ρ_)	0.95 - 1.74% chip-specific factor
Beam quality correction (k_Q_)	<1% for MV photons and electrons for lower energies, see Figure 4 and Table 1
Angular dependence (k_Θ_)	Not detected beyond intrinsic uncertainties in the system

## Discussion

This characterization of the measurement uncertainty of the myOSLchip system for IVD focused on energy dependence, dose linearity, repeat measurements, time to readout, and angular dependence. In general, when accounting for all factors contributing to measurement uncertainty, the dosimetric accuracy of the chip readout appears to be within one standard deviation of less than 1%. Multi-reader evaluation of dose linearity up to doses of 10 Gy demonstrated R^2^ values ≥0.9976 for all readers. As noted in the results, reader B demonstrated larger uncertainties than readers A or C due to a manufacturing defect that required the reader to be replaced. This linearity is comparable to the work of Kowalski et al. [6], who demonstrated linearity within 3% for a dose range of 0.1-20 Gy.

If performing a simple repeat measurement of an irradiated chip, we found that the signal will deteriorate on average by 1.33% per repeat reading. A previous report on the myOSLchip BeO chips had shown a signal loss of about 2% per repeat measurement [11], which agrees well with our findings. The time to readout, or dose fading, was found to be a critical factor influencing the accuracy of dosimetry readings, with 4 hours identified as the minimum time for the readout to be stable. Performing the readout as early as 10 min following irradiation or within 30-60 min could result in a dose reading that is ~4.5% or 2-3%, respectively, higher compared to the readout at 4 or 16 hours following radiation exposure. Conversely, there was no impact on dosimetric accuracy from reading the chips at later time points, with measurements up to 24 hours post-irradiation yielding the same results as at 4 hours, which agrees with a previous report on OSLDs of a different variety [12]. 

Repeat use of the myOSLchip can facilitate resource sparing by bleaching and re-using the chips after irradiation and readout. As demonstrated here, there is clearly a continuous drop in signal with repeated bleaching cycles that would need to be corrected for. Even when applying such a correction, there is considerable uncertainty about how individual chips respond to bleaching, and one should expect an added uncertainty of about 2% based on the standard deviation seen in Figure 3, in addition to the average 0.35% drop in signal per bleaching cycle. A recent report on a different OSL chip system suggested that pre-irradiation and bleaching with light not containing UV could create a more robust setting for reusing chips, with an approximate 1.4% uncertainty [13].

As clearly demonstrated, there is a strong energy dependence for these BeO chips at photon energies below the MV range, which has also been demonstrated previously [6,11]. Above the MV range, there was no noticeable energy dependence, and as such, a calibration performed at e.g. 6 MV would also apply to other clinical energies in the MV range. This also does not preclude the use of the myOSLchip for in-vivo dosimetry for systems employing lower energies, it would simply require an adjustment to the reading based on the characterized under response at the given energy.

Angular dependence was also investigated previously, and similarly to our findings, there was no systematic angular dependence but rather a mix of increased and decreased readings at different angles [6]. Similarly, the authors did not find any dependence on dose rate, something we did not investigate as part of this study.

A limitation of this study is that during the testing phase, another report by Kowalski et al. was independently published, focusing on similar work evaluating the myOSLchip system, and as such, this report should be viewed as confirmatory of their work. Furthermore, this report represents testing performed at a single institution and, as a result, it may not be representative of general in vivo dosimetry practices. This work still offers a valuable perspective for the field, both by independently validating previous findings and also by expanding on key aspects such as energy dependence and multi-reader evaluation.

## Conclusions

The myOSLchip BeO chip system for IVD enables accurate measurements well within the generally accepted clinical accuracy of ±3-5% for such readings. This is, of course, dependent on following proper calibration protocols such as determining chip-specific calibration factors for a given reader, ensuring a minimum of four hours until readout, and being aware of the number of repeat irradiations of a given chip and the effect this has on measurement accuracy.
